# 液液提取-固相萃取-高效液相色谱-串联质谱测定人体血液中16种有机磷酸酯

**DOI:** 10.3724/SP.J.1123.2020.07033

**Published:** 2021-01-08

**Authors:** Minmin HOU, Yali SHI, Yaqi CAI

**Affiliations:** 1.中国科学院生态环境研究中心, 环境化学与生态毒理学国家重点实验室, 北京 100083; 1. State Key Laboratory of Environmental Chemistry and Ecotoxicology, Research Center for Eco-Environmental Science, Chinese Academy of Sciences, Beijing 100083, China; 2.中国科学院大学, 北京 100049; 2. University of Chinese Academy of Sciences, Beijing 100049, China

**Keywords:** 固相萃取, 液液提取, 高效液相色谱-串联质谱, 有机磷酸酯, 人体血液, solid phase extraction (SPE), liquid-liquid extraction (LLE), high performance liquid chromatography-tandem mass spectrometry (HPLC-MS/MS), organophosphate ester, human blood

## Abstract

人体体液中有机磷酸酯(OPEs)浓度的测定对于了解人体OPEs的暴露水平以及评估人体健康风险具有重要意义。然而,目前的研究大多数集中于尿液中OPEs代谢物含量的分析测定,将其作为人体OPEs暴露的生物标志物,而对人体血液中OPEs的分析研究较少,仅有的少量研究涉及的OPEs种类有限。该研究在优化前处理过程(固相萃取,SPE)和色谱分离的基础上,建立了人体血液中16种OPEs的超高效液相色谱-串联质谱(UPLC-MS-MS)测定方法。血液样品经过乙腈摇床萃取后,经ENVI-18 SPE小柱净化,然后采用Acquity UPLC BEH C18色谱柱,以甲醇/5 mmol/L的乙酸铵水溶液为流动相进行梯度洗脱对目标物进行分离,最后进行LC-MS/MS测定。质谱分析采用电喷雾正离子模式电离,多重反应监测模式测定,内标法定量。在优化的检测条件下,16种OPEs的检出限为0.0038~0.882 ng/mL。除磷酸三甲酯(TMP)外,其余15种OPEs在3个浓度水平的血液基质加标回收率为53.1%~126%,相对标准偏差为0.15%~12.6%。样品的基质效应检测发现,4种OPEs存在明显的基质抑制,选用合适的同位素内标进行定量,可以部分消除基质影响。该方法样品前处理简单,灵敏度高,适用于人体血液样品中OPEs阻燃剂的测定。15个人体血液样本分析结果表明,OPEs的总浓度范围为1.50~7.99 ng/mL,其中8种OPEs的检出率均高于50%,磷酸三异丁酯(TiBP)、磷酸三(2-氯乙基)酯(TCEP)和磷酸三(1-氯-2-丙基)磷酸酯(TCIPP)为主要的OPEs,表明人体存在较为普遍的OPEs暴露,应该引起关注。

## Abstract (en)

Measurement of organophosphate esters (OPEs) in human body fluids is important for understanding human internal exposure to OPEs and for assessing related health risks. Most of the current studies have focused on the determination of OPE metabolites in human urine, as OPEs are readily metabolized into their diester or hydroxylated forms in the human body. However, given the existence of one metabolite across multiple OPEs or multiple metabolites of one OPE, as well as the low metabolic rates of several OPEs in *in vitro* studies, the reliability of urinary OPE metabolites as biomarkers for specific OPEs is needs to be treated with caution.Human blood is a matrix that is in contact with all body organs and tissues, and the blood levels of compounds may better represent the doses that reach target tissues. Currently, only a few studies have investigated the occurrence of OPEs in human blood by different analytical methods, and the variety of OPEs considered is limited. In this study, a method based on liquid chromatography-tandem mass spectrometry (LC-MS/MS) was developed for the simultaneous determination of 16 OPEs in human blood, and the extraction efficiency of the solid phase extraction (SPE) column for OPEs was verified. To human blood samples, 10 ng of an internal standard was added, followed by mixing and aging for 30 min. The samples were extracted three times with acetonitrile using a shaker, and then purified on ENVI-18 cartridges with acetonitrile containing 25% dichloromethane as the eluent. Finally, the OPEs were analyzed by high performance liquid chromatography-tandem mass spectrometry. After optimization of the analytical column and mobile phases, the analytes were separated on a BEH C18 column (100 mm×2.1 mm, 1.7 μm) by gradient elution using methanol and 5 mmol/L ammonium acetate in water as the mobile phase. Then, the analytes were ionized in electrospray ionization positive (ESI^+^) mode and detected in the multiple reaction monitoring (MRM) mode. The mass spectral parameters, including the precursor ion, product ion, declustering potential, entrance potential, and collision cell exit potential, were optimized. The results were quantified by the internal standard method. The limits of detection (LOD, *S/N*=3) of the OPEs were in the range of 0.0038-0.882 ng/mL. The calibration curves for the 16 OPEs showed good linear relationships in the range of 0.1-50 ng/mL, and the correlation coefficients were >0.995. The extraction efficiency of the ENVI-18 column for the 16 OPEs was validated, and the average recoveries of the target compounds were 54.6%-104%. The average recoveries (*n*=3) of 15 OPEs, except trimethyl phosphate (TMP), in whole blood at three spiked levels were in the range of 53.1%-126%, and the relative standard deviations (RSDs) were in the range of 0.15%-12.6%. The average recoveries of six internal standards were in the range of 66.8%-91.6% except for TMP-d9 (39.1%), with RSDs of 3.52%-6.85%. The average matrix effects of the OPEs in whole blood were 56.4%-103.0%. Significant matrix effects were found for resorcinol bis(diphenyl phosphate) (RDP) (75.8%±1.4%), trimethylphenyl phosphate (TMPP) (68.4%±1.0%), 2-ethylhexyl di-phenyl phosphate (EHDPP) (56.4%±12.4%), and bisphenol-A bis(diphenyl phosphate) (BABP) (58.5%±0.4%). However, these effects could be corrected by similar signal suppressions of the corresponding internal standard (TPHP-d15, 77.4%±7.5%). This method is simple, highly sensitive, and suitable for the determination of OPEs in human blood. Fifteen human whole blood samples were collected to quantify the 16 OPEs using the developed method. The total concentrations of the OPEs ranged from 1.50 to 7.99 ng/mL. The detection frequencies of eight OPEs were higher than 50%. Tri-iso-butyl phosphate (TiBP), tri(2-chloroethyl) phosphate (TCEP), and tri(1-chloro-2-propyl) phosphate (TCIPP) were the dominant OPEs, with median concentrations of 0.813, 0.764, and 0.690 ng/mL, respectively. These results indicated widespread human exposure to OPEs, which should be of concern.

近几年,多溴联苯醚(PBDEs)因具有持久性、长距离迁移性、生物累积性以及毒性而在世界范围内被禁止使用并且逐渐退出市场,有机磷酸酯(OPEs)作为其优良的替代品,生产量和使用量显著增加,作为阻燃剂和增塑剂广泛应用于泡沫、塑料、纺织制品以及液压油和各种建材产品中^[1,2,3]^。2015年,OPEs的全球使用量高达68万吨,年使用增长率约为7.9%^[4]^。OPEs通过物理的方式添加进各种消费品中,因此很容易通过挥发,磨损或者渗滤的方式释放进入到环境中^[1]^。目前,已经有大量研究在大气^[5,6]^、水体^[6,7,8]^、土壤^[9]^、沉积物^[7,10]^、灰尘^[11,12]^等多种环境介质以及生物体^[8,13]^中检出OPEs。此外,毒理学研究已经证实部分OPEs的暴露可能会对人体及其他生物体造成不良影响,包括致癌性^[14]^、神经毒性^[15]^、生殖毒性^[16]^、甲状腺激素^[17]^和雌激素干扰效应^[18]^、哮喘以及过敏性鼻炎^[19]^等。

环境介质中的OPEs可通过呼吸、灰尘摄食、真皮吸收或者饮食进入人体,进而对人体健康造成危害。目前国内外已经有较多的研究在人体尿液^[20,21]^、血液^[22,23,24,25]^、头发^[26,27,28]^、指甲^[27]^以及母乳^[29,30]^等样品中检测到OPEs的存在,表明了普遍的人体OPEs的暴露。进入人体内的OPEs很容易代谢成其二酯类或者羟基类的化合物,进而通过尿液排出体外^[31,32,33,34]^。因此,目前大多数研究主要集中于尿液中OPEs代谢物的检测,将其作为人体OPEs暴露的生物标志物^[35]^。然而,有些OPEs,如磷酸三(2-氯乙基)酯(TCEP),在人体内的代谢速率较慢^[31]^。此外,尿液中某一种OPEs的代谢物可能是由多种不同的OPEs代谢产生,如磷酸三苯酯(TPHP)、2-乙基己基二苯磷酸酯(EHDPP)和间苯二酚双(磷酸二苯酯)(RDP)均可以代谢产生磷酸二苯酯(DPHP)^[31,36-38]^。并且,有些OPEs二酯代谢物,如磷酸二(2-乙基己基)酯(DEHP)、磷酸二丁酯(DnBP)和DPHP,有直接的生产和使用,并且已有研究在室内灰尘和食品中检测出它们的存在^[39,40]^,表明这些物质可能会直接暴露于人体。因此,对于某些OPEs,相比尿液中的代谢物,血液中母体物质的检测可能更能准确反映人体对于OPEs的暴露。且因人体内血液与各个器官和组织直接接触,血液中化合物的浓度更能反映到达特定组织的剂量,进而更准确地评估人体健康风险。

目前,已有少量研究检测了人体血液中OPEs的存在,通过使用不同的分析检测方法,包括固相萃取(SPE)联用GC-MS^[41,42,43]^和液液提取-双SPE柱固相萃取和LC-MS/MS联用^[22,25]^。但是,这些研究所检测的OPEs种类相对较少。另外,随着工业和科学研究的不断推进,不断有结构性能各异的OPEs新产品被大量生产和使用,近几年已经有较多新型的OPEs在各种消费品及其相关环境中检出。因此,建立同时检测人体血液样品中多种OPEs的分析方法具有重要意义和迫切需求。本工作针对16种OPEs,通过优化SPE等前处理方法和色谱-质谱方法,建立了灵敏高效的同时检测人体血液中多种OPEs的高效液相色谱-串联质谱分析方法,为研究人体OPEs的暴露水平和积累特征提供方法基础。

## 1 实验部分

### 1.1 仪器、试剂与材料

Ultimate 4500液相色谱仪及Triple quad^TM^ 4500三重四极杆质谱仪(MS/MS,美国AB SCIEX公司),系统配有电喷雾(ESI)离子源和Analyst 1.6.2工作站;氮吹浓缩仪;ENVI-18 SPE小柱(6 mL, 500 mg; Supelco)。

甲醇、乙腈(色谱纯,美国Merck公司);二氯甲烷(色谱纯,美国Fisher公司); Milli-Q超纯水制备系统(美国Millipore公司)。

16种目标分析物信息如表1所示,其中TMP、TEP、TPrP、TnBP、TiBP、TEHP、TBOEP、TCEP、TCIPP、TDCPP、TPHP、TMPP、EHDPP和CDPP购自德国Dr. Ehrenstorfer公司;RDP、BABP、TCIPP-d18和TCEP-12购自加拿大Toronto Research Chemicals公司;内标TMP-d9、TEP-d15和TPrP-d21购自加拿大C/D/N Isotopes公司;TnBP-d27和TPHP-d15购自美国Cambridge Isotope Laboratories公司。

**表 1:** 16种OPEs的英文全称、简称、分子式、相对分子质量及CAS号

Compound	Abbreviation	Formula	M_r_	CAS No.
Trimethyl phosphate	TMP	C_3_H_9_O_4_P	140.08	512-56-1
Triethyl phosphate	TEP	C_6_H_15_O_4_P	182.16	78-40-0
Tripropyl phosphate	TPrP	C_9_H_21_O_4_P	224.23	513-08-06
Tri-n-butyl phosphate	TnBP	C_12_H_27_O_4_P	266.31	126-73-8
Tri-iso-butyl phosphate	TiBP	C_12_H_27_O_4_P	266.31	126-71-6
Tris(2-ethylhexyl) phosphate	TEHP	C_24_H_51_O_4_P	434.63	78-42-2
Tri(2-butoxyethyl) phosphate	TBOEP	C_18_H_39_O_7_P	398.47	78-51-3
Tri(1-chloro-2-propyl) phosphate	TCIPP	C_9_H_18_Cl_3_O_4_P	327.57	13674-84-5
Tri(2-chloroethyl) phosphate	TCEP	C_6_H_12_Cl_3_O_4_P	285.49	115-96-8
Tri(1,3-dichloro-2-propyl) phosphate	TDCPP	C_9_H_15_Cl_6_O_4_P	430.90	13674-87-8
Tri-phenyl phosphate	TPHP	C_18_H_15_O_4_P	326.28	115-86-6
Trimethylphenyl phosphate	TMPP	C_21_H_21_O_4_P	368.36	563-04-2
Cresyl diphenyl phosphate	CDPP	C_19_H_17_O_4_P	340.31	26444-49-5
2-Ethylhexyl di-phenyl phosphate	EHDPP	C_20_H_27_O_4_P	362.41	1241-94-7
Resorcinol bis(diphenyl phosphate)	RDP	C_30_H_24_O_8_P_2_	574.45	57583-54-7
Bisphenol-A bis(diphenyl phosphate)	BABP	C_39_H_34_O_8_P_2_	692.63	5945-33-5

血液样本:采集对象为山东省济南市的15名健康老年人,所有参与志愿者在采样前均详细阅读并签署了知情同意书。

### 1.2 血液样品前处理

参考文献^[44]^的方法进行样品前处理(略有修改),并进行验证。具体过程如下:血液解冻后取0.5 mL于15 mL玻璃离心管中,加入10 μL内标混合溶液(1 ng/μL),涡旋混匀后静置30 min,再加入10 mL乙腈,摇床萃取12 h,离心后将上清液转移至另一个干净离心管中;再向残余部分加入2 mL乙腈,按照上述步骤重复萃取两次,每次30 min,最后将3次萃取所得上清液合并,氮吹浓缩至约0.5 mL,加入30 mL超纯水稀释待净化。考虑到部分OPEs物质容易挥发,氮吹过程中氮气流速以液面轻微波动即可,氮吹温度为50 ℃。

依次用5 mL乙腈和5 mL超纯水活化ENVI-18小柱,将萃取液加载到活化好的小柱上,上样完成后先用10 mL的超纯水清洗小柱;清洗液流干后,在负压下对小柱抽干约40 min,之后用6 mL含有25%二氯甲烷的乙腈进行洗脱,洗脱液氮吹至近干,甲醇定容至1 mL,通过0.22 μm的有机滤膜后进行UPLC-MS/MS测定。

### 1.3 仪器检测条件

色谱 色谱柱Acquity UPLC BEH C18柱(100 mm×2.1 mm, 1.7 μm),连接保护柱Acquity UPLC BEH C18(5 mm×2.1 mm);流动相:A为5 mmol/L醋酸铵缓冲溶液,B为甲醇(MeOH);柱温25 ℃,流速400 μL/min;梯度洗脱程序为:0~1 min, 10%B~40%B; 1~4 min, 40%B~90%B; 4~4.1 min, 90%B~100%B,维持4.9 min; 9~9.1 min, 100%B~10%B,维持3.9 min。

质谱 电喷雾离子源(ESI),正离子多重反应监测(MRM)模式;针泵进样,在确定母离子和子离子对后,对解簇电压(DP)、入口电压(EP)、碰撞电压(CXP)等参数进行优化(见表2)。接入色谱流动相后,对其他参数进行优化,结果如下:气帘气压为0.14 MPa,碰撞气压为0.02 MPa,离子源喷雾电压为5000 V,温度为600 ℃,雾化气为0.34 MPa,辅助雾化气为0.28 MPa。

**表 2:** 16种OPEs的质谱参数

Analyte	Precursor ion (m/z)	Product ion (m/z)	Declustering potential (DP)/V	Entrance potential (EP)/V	Collision cell exit potential (CXP)/V
TMP	141.1	109.1^*^	60	22	10
		79.0	60	29	6
TEP	183.0	99.0^*^	54	24	7
		81.0	60	50	8
TPrP	225.4	99.0^*^	60	22	7
		141	60	24	10
TnBP	267.4	99.0^*^	60	20	10
		155	60	12	10
TiBP	267.4	99.0^*^	60	20	10
		155	60	12	10
TEHP	435.3	99.0^*^	140	22	9
		113.1	120	16	8
TBOEP	399.3	299.3^*^	95	19	10
		199.0	95	21	10
TCIPP	327.0	99.0^*^	70	30	10
	329.1	99.0	70	28	10
TCEP	285.0	63.0^*^	80	42	10
		99.2	75	30	10
TDCPP	431.1	98.9^*^	85	35	9
		208.9	84	20	8
TPHP	327.1	152.0^*^	130	42	11
		77.1	130	65	7
TMPP	369.2	166.1^*^	147	37	11
		90.9	147	61	8
CDPP	341.1	152.1^*^	135	40	10
		165.1	135	40	10
EHDPP	363.2	76.9^*^	70	71	7
		251.0	72	12	9
RDP	575.2	419.2^*^	190	46	15
		481.1	183	46	15
BABP	693.2	367.0^*^	200	45	15
		693.3	200	12	15
TMP-d9	150.1	83.1	90	31	7
TEP-d15	198.1	101.9	65	27	8
TPrP-d21	246.4	102	120	25	9
TCEP-d12	299.1	102	75	30	6
TnBP-d27	294.4	101.9	140	25	10
TPHP-d15	342.3	160	135	47	10
TCIPP-d18	345.1	101.9	75	30	8

* Quantitative ions.

## 2 结果与讨论

### 2.1 SPE柱的回收率

通过在ENVI-18 SPE柱上加载30 mL含100ng OPEs和10 ng内标的超纯水溶液,考察了该SPE柱对16种目标OPEs的回收率,16种OPEs的回收率为54.6%~104%(见表3), 7种内标TMP-d9、TEP-d15、TPrP-d21、TCIPP-d18、TCEP-12、TnBP-d27和TPHP-d15的回收率分别为61.3%±5.04%、70.8%±5.49%、99.1%±8.06%、113%±3.09%、98.9%±6.95%、96.6%±5.15%和95.9%±2.90%,该回收率满足物质分析的需求。

**表 3:** ENVI-18 SPE柱对16种OPEs的提取回收率

Analyte	Recoveries/%				RSD/%
	1	2	3	Mean	
TMP	52.7	59.3	51.6	54.6	4.2
TEP	63.0	68.8	67.3	66.3	3.0
TPrP	79.5	79.5	84.3	81.1	2.7
TnBP	76.2	77.2	80.5	78.0	2.3
TiBP	77.6	80.3	78.5	78.8	1.4
TEHP	73.3	78.8	74.9	75.7	2.8
TBOEP	81.4	89.3	94.9	88.5	6.8
TCEP	80.8	88.1	73.7	80.9	7.2
TCIPP	87.9	91.5	87.9	89.1	2.1
TDCPP	66.3	74.8	70.8	70.6	4.2
TPHP	82.6	83.0	81.4	82.3	0.8
EHDPP	69.7	90.5	81.8	80.7	10
TMPP	76.8	83.0	82.6	80.8	3.5
CDPP	101	106	104	104	2.6
RDP	85.1	92.0	92.3	89.8	4.1
BABP	79.1	82.3	89.2	83.5	5.1

### 2.2 色谱柱和流动相的选择

本研究考察了Acclaim Mixed-Mode HILIC-1(150 mm×2.1 mm, 5 μm; Thermo Fisher)和Acquity UPLC BEH C18(100 mm×2.1 mm, 1.7 μm; Waters)2种类型的色谱柱对16种OPEs的分离和保留能力。Mixed-Mode HILIC-1色谱柱的固定相由疏水性烷基链组成,末端是二醇基团,这使其既具有疏水保留,又具有亲水相互作用。BEH C18柱作为一种通用的C18色谱柱,适用于各种分析物的分离。比较结果表明,2种色谱柱均能对目标化合物实现较好的分离,但个别疏水性的OPEs如TEHP等的分离,相比C18柱,它们在Acclaim Mixed-Mode HILIC-1柱上的保留时间较长,考虑到分析效率,本研究最终采用UPLC BEH C18色谱柱进行16种OPEs的UPLC-MS/MS分析。

比较UPLC BEH C18色谱柱在甲醇-2 mmol/L、5 mmol/L、50 mmol/L乙酸铵水溶液3种流动相组成条件下对16种OPEs分离的峰形和灵敏度,结果表明,当采用5 mmol/L的乙酸铵水溶液时,大多数OPEs的响应略高且获得良好分离。因此,本研究最终采用甲醇-5 mmol/L乙酸铵水溶液作为流动相。16种OPEs标准溶液的总离子流色谱图见图1。

**图 1:**
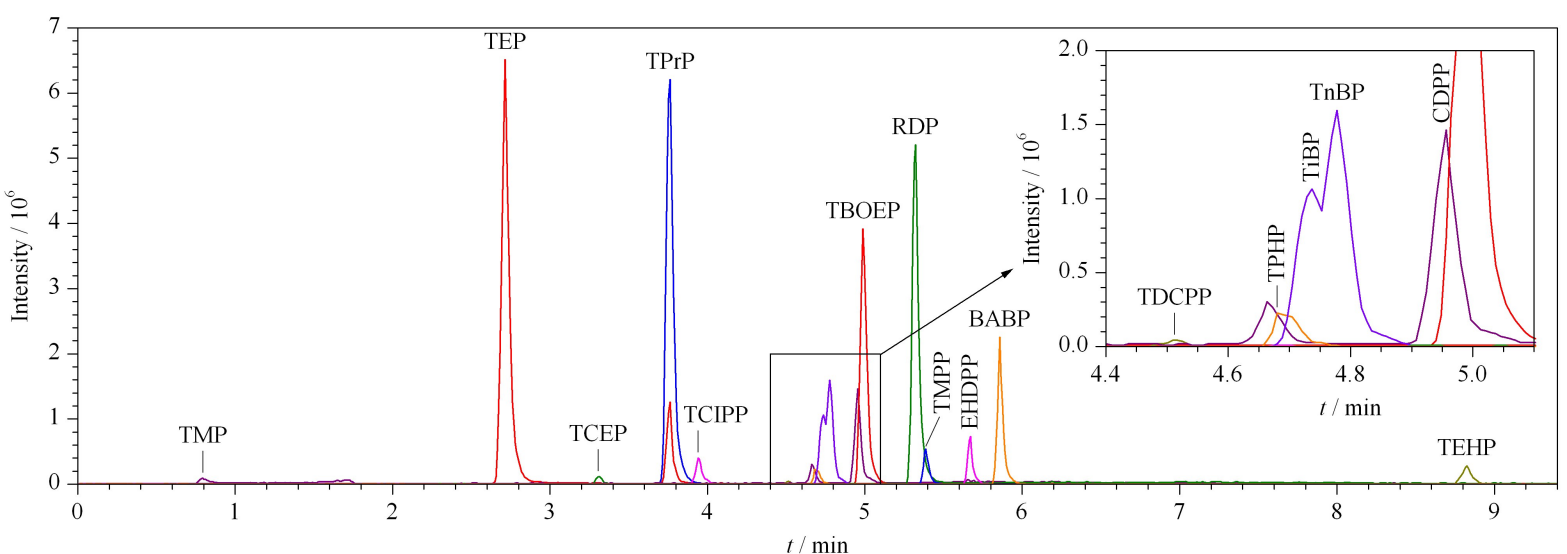
16种OPEs标准品的总离子流色谱图

### 2.3 方法学表现

按照优化后的检测条件测定0.1、0.5、2、5、10、20、50 ng/mL的混合标准溶液,以目标物质与内标的浓度比为横坐标,峰面积比为纵坐标进行线性回归,结果表明,16种OPEs在0.1~50 ng/mL范围内有良好的线性关系,线性相关系数均在0.995以上。检出限(LOD)以3倍信噪比计算,结果见表4, 16种OPEs的LODs为0.0038~0.882 ng/mL。

**表 4:** 16种OPEs在人体血液中3个水平下的加标回收率(*n*=3)

Analyte	Spiked 2 ng/mL			Spiked 20 ng/mL			Spiked 40 ng/mL		LOD/(ng/mL)
	Recovery/%	RSD/%		Recovery/%	RSD/%		Recovery/%	RSD/%	
TMP	30.6	2.2		33.3	1.0		37.3	6.0	0.83
TEP	72.6	5.7		70.6	6.6		71.1	3.8	0.17
TPrP	82.2	1.9		78.5	1.4		70.8	1.4	0.038
TnBP	88.1	7.6		78.6	3.0		71.0	2.9	0.042
TiBP	79.9	12.3		70.6	8.4		77.3	10.4	0.020
TEHP	63.6	7.9		53.1	12.3		72.7	12.6	0.070
TBOEP	85.9	4.9		84.3	5.7		77.8	3.0	0.015
TCIPP	72.1	4.7		79.9	4.2		74.2	2.9	0.26
TCEP	79.4	3.5		71.6	7.0		70.8	1.4	0.26
TDCPP	64.9	8.9		70.6	4.2		74.3	1.8	0.15
TPHP	75.4	1.0		77.1	10.4		75.7	4.5	0.15
TMPP	60.2	1.4		65.2	0.71		65.5	0.15	0.028
CDPP	126	12.1		114	5.2		111	6.5	0.88
EHDPP	58.1	2.2		55.0	6.8		68.5	3.6	0.034
RDP	69.1	4.7		62.1	4.7		64.1	2.0	0.024
BABP	63.5	8.6		67.6	3.7		63.5	3.9	0.0038

为了考察方法的有效性和精密度,进行了基质加标回收试验。将之前采集的几个志愿者的血液样品混合,作为基质。分别取0.5 mL的血液基质,加入2、20、40 ng/mL的混合标准溶液,每个浓度水平进行3次重复试验,按照优化的样品前处理和检测条件进行实验。结果见表4,除TMP外,其余15种OPEs的基质加标回收率为53.1%~126%,相对标准偏差为0.15%~12.6%。对于TMP,采用其氘代同位素TMP-d9作为内标物对TMP在样品处理过程中的损失进行校正,TMP-d9的加标回收率为39.1%±3.97%。其余6种内标的加标回收率为66.8%±6.85%~91.6%±3.52%。

### 2.4 样品基质效应评估

本研究采用提取后添加法评估人体血液样本的基质效应(ME),具体过程如下:分别取6个血液样本,不添加任何目标物质以及内标,根据样品前处理过程进行萃取净化,将最终获得的萃取液进行混合(6 mL)作为基质空白。取1 mL空白基质加入10 ng的标准物质和内标,按照样品检测方法测定,获得加标基质响应(*A*)。另取1 mL空白基质,不加任何标准上机去检测,获得空白基质响应(*B*), *C*为纯溶剂中相同浓度待测物质的响应。进行3个平行,目标化合物的基质效应通过如下公式计算:

ME= $\frac{A-B}{C}$ ×100%

结果表明,人体血液样本中待测的16种OPEs的基质效应为56.4%±12.4%~103.0%±1.1%。其中,TCEP(88.6%±1.3%)和TCIPP(77.5%±4.3%)存在较弱的基质抑制,可以通过其相应的同位素内标(TCEP-d12(75.3%±8.9%)和TCIPP-d18(77.4%±7.5%))进行消除。此外,RDP、TMPP、EHDPP和BABP存在明显的基质抑制,分别为75.8%±1.4%、68.4%±1.0%、56.4%±12.4%和58.5%±0.4%,这4种待测物质没有相应的同位素内标。本研究采用内标法定量,使用TPHP-d15(77.4%±7.5%)作为它们的内标进行定量,可以部分消除基质效应的影响,满足分析要求。

### 2.5 实际样品测定

使用本研究建立的分析检测方法,对采集的15个人体血液样本中的OPEs进行分析测定,结果见表5。16种OPEs的总浓度为1.50~7.99 ng/mL,除TMP、TEP、TPrP、CDPP、TMPP、BABP、RDP和TDCPP外,其余8种OPEs检出率均高于50%。其中TiBP、TCEP和TCIPP的中位浓度最高,分别为0.813 ng/mL、0.764 ng/mL和0.690 ng/mL。

**表 5:** 人体血液样本的分析结果

No.	TMP	TEP	TPrP	TnBP	TiBP	TEHP	TBOEP	TPHP	EHDPP	CDPP	TMPP	BABP	RDP	TCEP	TCIPP	TDCPP	∑_16_OPE
1	-	0.160	-	-	0.235	-	0.793	0.462	0.485	-	-	0.029	-	2.490	0.290	-	4.94
2	-	-	-	-	0.960	-	-	0.332	-	-	-	-	-	1.378	0.590	-	3.26
3	-	-	-	-	0.378	0.115	-	0.335	0.165	-	-	-	-	2.415	0.415	-	3.82
4	-	-	-	-	-	0.178	-	0.392	0.153	-	-	-	-	-	1.615	-	2.34
5	-	-	-	-	0.953	0.290	0.245	0.390	0.230	-	-	0.023	-	0.298	0.765	0.460	3.65
6	-	-	-	0.225	-	0.398	0.185	0.270	0.155	-	-	-	-	0.523	-	-	1.76
7	-	-	-	0.218	0.920	0.138	-	-	0.048	-	-	-	0.132	-	0.240	-	1.69
8	-	0.118	-	-	0.365	0.163	0.238	0.252	0.070	-	-	-	-	-	0.640	-	1.85
9	-	-	-	0.673	1.413	-	-	0.485	0.123	-	-	-	-	-	1.440	-	4.13
10	-	0.263	-	1.173	1.488	0.375	0.713	0.330	0.128	0.795	-	-	-	0.708	2.015	-	7.99
11	-	-	-	0.538	0.803	-	1.010	0.910	0.280	-	-	-	-	0.820	2.140	-	6.50
12	-	0.115	-	0.200	0.313	0.878	0.750	0.151	0.210	-	-	0.006	-	-	0.165	-	2.79
13	-	-	-	1.505	1.693	0.363	0.410	-	0.325	-	-	0.007	-	0.628	2.365	0.538	7.83
14	-	-	-	-	0.270	-	0.153	0.335	-	-	-	0.005	-	-	0.740	-	1.50
15	-	-	-	0.230	0.813	0.365	0.598	-	0.383	-	-	-	-	-	0.415	-	2.80

-: not detected.

## 3 结论

本工作建立了人体血液中16种有机磷酸酯阻燃剂的UPLC-MS/MS检测方法,该方法仅需要0.5 mL血液样品,前处理流程操作简便,目标化合物和内标物获得良好的回收率,检测灵敏度高,重现性好,可以满足人体血液中OPEs的检测要求。
